# Parents’ perception of adolescents’ difficulties and impact of problems in different castes and ethnic groups in Nepal. Do they converge with the frequencies of symptoms reported on the child behavior checklist (CBCL)?

**DOI:** 10.1007/s00127-025-02835-1

**Published:** 2025-02-17

**Authors:** Sirjana Adhikari, Jasmine Ma, Suraj Shakya, Per Håkan Brøndbo, Bjørn Helge Handegård, Anne Cecilie Javo

**Affiliations:** 1 Department of Psychology, Faculty of Health Sciences, Uit, the Arctic University of Norway, Tromsø, Norway; 2 CWIN-Nepal, Ravi Bhawan, Kathmandu, Nepal; 3 Department of Psychiatry and Mental Health, Institute of Medicine, Tribhuvan University, Kathmandu, Nepal; 4 Faculty of Health Sciences, Regional Centre for Child and Youth Mental Health and Child Welfare - North, Uit, the Arctic University of Norway, Tromsø, Norway; 5 Sami National Competence Center for Mental Health (SANKS), Sami Klinihkka, Finnmark Hospital Trust, Karasjok, Norway

**Keywords:** SDQ, Perceived difficulties, Impact of problems, CBCL, Adolescent, Nepal

## Abstract

**Background:**

Parents’ perceptions of their children’s behavior are culturally determined and may differ across cultures. The present study aimed to investigate parents’ perceptions of adolescents’ difficulties and the impact of problems in different cultural contexts in Nepal, and to explore the extent to which they align with child symptoms measured on a problem rating scale.

**Methods:**

This study was conducted with parents of school-going adolescents in sixteen districts of Nepal. The Nepali version of the Strengths and Difficulties Questionnaire (SDQ)—Impact Supplement was used to assess parents’ perception of difficulties and the impact of problems, and the Child Behavior Checklist/6-18 (CBCL) was used as a symptom rating scale. We employed a mixed model approach for data analysis to address the hierarchical structure of our data.

**Results:**

Parents’ perceptions of difficulties and the impact of problems did not differ between the Hindu “high caste”, the Hindu “low caste” and the indigenous/ethnic minority group. In contrast, the effect of caste/ethnicity was significant for parent ratings on the CBCL Total Problems as the “low caste” parents reported higher mean scores than parents from the indigenous/ethnic minorities group. Parents’ perception of difficulties and the impact of problems were moderately associated with their reports on the CBCL Total Problems. There was no moderating effect of caste/ethnicity on any of these associations.

**Conclusion:**

Although cross-cultural differences emerged in parents’ ratings of symptoms, no differences emerged in their perception of difficulties and the impact of problems. Moderate associations between the CBCL Total Problems and perceived difficulties and the impact of problems suggest that clinicians should consider using supplement measurements in their assessment of child behavior problems. However, further studies are required to confirm our findings.

**Supplementary Information:**

The online version contains supplementary material available at 10.1007/s00127-025-02835-1.

## Introduction

Parents’ perception of children’s difficulties and the degree of impairment can vary based on cultural and socioeconomic factors [1–3]. Recent studies that have examined the link between culture and child behavior, emphasize the importance of investigating the cultural meanings of adaptive and maladaptive behaviors [4]. Problem behaviors in one ecocultural setting may have different significance and substantially different meanings in other settings [5]. Hence, parents in one culture might have different perceptions about the threshold for labeling a child’s behavior as deviant from parents in another culture, even if their children have similar problems [2, 6–8]. There may be cultural differences in coping, adaptation, and concern for children’s behavioral problems [5, 9]. Weisz et al. explained the importance of context-dependent meanings of mental symptoms: “Child psychopathology is inevitably the study of two phenomena — the behavior of children and the lens through which adults view child behavior” [10]. Previous cross-cultural research has consistently shown variations in parents’ ratings of children and adolescents’ emotional and behavioral problems (EBPs), indicating that they might be partly influenced by cultural norms [8, 11–14]. Recent studies have confirmed these findings [15–18]. In Asian cultures, cultural norms and values such as behavioral conformity, obeying adults, interdependence among family members, and inhibition of inner impulses have been emphasized [19, 20]. Hence, Asian parents may tend to perceive withdrawn- or internalizing behaviors in children and adolescents as less serious and worrisome as they might be more in tune with their own cultural norms and socialization goals [7].

When measuring EBPs cross-culturally, it should be noted that the frequency of behaviors reported on problem rating scales such as the Child Behavior Checklist (CBCL), may not always align with parents’ perception of difficulties. While screening instruments are valuable for obtaining a broad overview of child- and adolescent functioning by assessing the frequency of problem behaviors as reported by parents [21], they may not directly indicate whether a specific behavior is problematic. Parents might not necessarily perceive behaviors rated on such scales as burdensome to the environment or warranting interventions. Studies have shown that the degree of impairment associated with behaviors or mental health symptoms may vary based on cultural or socioeconomic factors [1]. Therefore, it is recommended to complement symptom rating scales with additional assessment procedures to evaluate parental perception and impact of problems as this approach ensures a more comprehensive understanding of the child’s difficulties and facilitates more effective intervention strategies [22, 23]. Measuring the impact of these problems may result in more accurate assessment and screening. Studies have demonstrated higher rates of disorders when symptoms alone are considered, and these rates are significantly reduced when impact is measured [1]. Consequently, including impairment ratings when screening for child difficulties reduces false positives and contributes to an efficient resource allocation. Compared to the Total Difficulties score on the Strengths and Difficulties Questionnaire (SDQ), the Impact Supplement was found to be a better predictor of psychiatric caseness [24, 24] and service use [26]. Furthermore, the measurement of impact may also help with the issue of under-identification of children at risk given that several studies have identified groups of children who are impaired without exhibiting clinical levels of symptoms, both in clinic-referred samples [27, 28] and in non-referred samples [29, 30]. The measurement of impact in addition to symptom ratings in a screening context allows the detection of sub-clinical difficulties and the identification of as many children as possible who are at risk [23].

Despite the clear benefits of measuring parent perceptions and the impact of problems in addition to symptoms, Nepali studies on EBPs have yet to incorporate such measures in their assessments. There has been a modest body of literature on culture and mental health in Nepal [31]), and few Nepali studies have compared potential cultural differences in parents’ perceptions and reports of problems, although Nepal is a multicultural country in terms of caste and ethnicity. The present study aimed to (1) assess and compare Nepali parents’ perceptions of adolescents’ overall difficulties and the impact of problems between different castes/ethnic groups, (2) compare parents’ ratings of symptoms on a symptom rating scale (CBCL) between the same castes/ethnic groups, (3) examine the association between parents’ perceptions of difficulties and the impact of problems, and their ratings on the CBCL Total Problems scale, and (4) examine the possible moderating effects of caste/ethnicity on any of the associations between the SDQ-impact of problems and the CBCL symptoms.

## Materials and methods

This study is part of a larger, cross-sectional survey of EBPs in school-going Nepali children and adolescents aged 6–18 years [32–34]. Two studies on the same sample, but limited to adolescents, have recently been published [35, 36].

### Study site and population

Nepal is a low-middle-income country (LMIC) with a Human Development Index of 0.60, placing it in the medium human development category [37]. About one-fourth of the people live below the poverty level, i.e., earn less than US$ 1.25 per day. The country is topographically divided into three regions: The Himalayas (mountain region), which constitutes 6.1% of the population, the Middle Hills region (40.3%); and the Terai region (53.6% of the population) [38]. Approximately one-third (34%) of the people live in rural areas. The remaining two-thirds (66%) live in the urban areas. Children up to 18 years of age constitute approximately 36% of the total population [38]. Adolescents’ social and health vulnerabilities are high in Nepal, especially among girls and those belonging to the “low caste” Hindu group [39]. The child mental health situation is difficult due to the absence of a child and adolescent mental health policy, few mental health services, and a shortage of specialized human resources [40].

#### Castes/Ethnicity

Nepal is a multicultural country with 142 castes/ethnic groups and 124 mother tongues [41]. The Chhetri group was the largest, accounting for 16.5% of the total population, followed by Brahmin-Hill (11.3%), Magar (6.9%), Tharu (6.2%), Tamang (5.6%), Khas Kaami (Dalit) 5.0%, and Newar 4.6% [41]. The Chhetri and Brahmin-Hill represent the Hindu “high castes”, and the Khas Kaami (Dalit) represent the Hindu “low caste”. In Nepal, the term “caste” basically refers to a group of people who follow Hinduism, speak Nepali or any other Indo-Aryan languages, and have been traditionally ranked according to the Hindu religious values of purity and impurity. Casteism is still practiced, especially in the rural communities of Nepal, despite the law declaring it illegal (the New Civil Code of 1963) [42]*.* The “high caste” groups enjoy a privileged status in society while the “low caste” group, who resides both in the Terai and Hilly regions, occupy the lowest social rung. The “low caste” group (the Dalits) may experience pervasive caste-based discrimination and disadvantages in various spheres of life [42, 43]. The next four groups, Magar, Tharu, Tamang, and Newar, all belong to indigenous minority groups collectively known as the “Janajati” or indigenous/ethnic minorities. They have indigenous traditions and languages, and constitute over one-third of Nepal’s population but live as a minority in all 77 districts of Nepal [41]. The caste-based social structure in Nepal is complex and further complicated by the fact that each ethnic group may have a hierarchical system with distinct socio-cultural norms and practices. For example, Newars have a well-defined hierarchically based occupational caste system that ranges from the lowest to the highest, from cleaner to priest [44].

### Participants and procedure

The participants were 1882 parents of school-going adolescents aged 11 to 18 years. Based on the population distribution of the three main ecological/geographical regions of Nepal, 16 districts (three districts from the mountain region, six districts from the Middle Hills region, six districts from the Terai region, and the Kathmandu district) and four schools (two government and two private) in each district were selected using a purposive sampling method. Given our nationwide study in a country with poor and time-consuming transportation and communication systems, the purposive sampling technique was chosen because of its cost-effectiveness and ease of data collection and travel. The children in each school were randomly selected using random number tables from Classes 6 to 10. Six students per class (3 boys and 3 girls) were selected. The data were collected in 2017 and 2018. The overall participation rate was 98.9%. The missing data were < 0.5% for each variable. More detailed information about the procedure has been provided in a previous paper [32]. The participants were categorized into three groups based on their caste/ethnicity, as described in a previous Nepali study [45]. According to the Nepal Census of 2011, all participants in the study belonged to the seven largest caste/ethnic groups [46]. The three main group categories were: (1) Brahmin and Chhetri (Hindu “high caste” group); (2) Janajati (indigenous/ethnic minorities group); and (3) Dalits (Hindu “low caste” group). Ethical approval was obtained from the Ethical Board of the Nepal Health Research Council (NHRC). A team of trained research assistants performed data collection and was monitored by the project leader. A meeting with the school management was conducted at each school, and an invitation letter was sent to the parents to participate in the study. Written informed consent was obtained from the parents of the selected students. Both verbal and written information about the study were provided to the parents. Parents were given the CBCL, the Impact Supplement of SDQ, and a background information questionnaire to report on problem behaviors, the impact of problems, and socio-demographic- and family background data. Parents first filled in the CBCL questionnaires and then completed the SDQ-Impact supplement questionnaires independently of the research assistants, except for illiterate parents who were assisted in filling in the forms.

### Measures

#### Impact supplement of the strengths and difficulties questionnaire (SDQ)

The main instrument used in the present study was the Nepali version of the “Impact Supplement of the extended Strengths and Difficulties Questionnaire” (SDQ) [47] elaborated by Goodman to explore the informant’s perception of a child’s difficulties, the impact of the difficulties in terms of distress and social impairment, and the burden to the family [23]. To measure parents’ perception of child difficulties, the first question of the SDQ Impact Supplement was used: "Overall, do you think that your child has difficulties in one or more of the following areas: emotions, concentration, behavior, or being able to get on with other people?” This question was scored on a 4-point scale: no, minor, definite, and severe. If the parents perceived “no difficulties,” items about the impact and burden of problems on the family were skipped and the impact was automatically scored as zero (no impairment). In the case of a positive response, the parents were further questioned about the impact of the problems. To assess distress, the question asked was: “Do the difficulties upset or distress your child?” A further question about the impact of problems was “Do the difficulties interfere with your child’s everyday life in the following areas: home life, friendships, classroom learning, and leisure activities?” The same options, i.e., “not at all”, “only a little”, “a medium amount”, and “a great deal” was provided for all four areas. The impact of problems was rated on a 4-point scale: 0 = not at all, 0 = only a little, 1 = quite a lot, and 2 = a great deal. The items concerning child distress (1 item) and social impairment (4 items) generated a total impact score ranging between 0 and 10 by adding the scores of all items using a “0-0-1–2” scale according to Goodman’s recommendation [23]. The SDQ impact score has high concurrent and predictive validity [25] and is acceptable for good internal consistency [25, 48].

#### Child behavior checklist (CBCL)

The CBCL/6–18 was used to assess adolescents’ EBPs, as reported by their parents. Written permission to use the Nepali version was granted by the copyright owner, which was made in connection with a Ph.D. dissertation [49]. The CBCL is a rating instrument commonly used worldwide and has been translated into more than 100 languages. It consists of 20 items measuring competencies and 120 items addressing behavioral problems and has established good psychometric properties [50]. The problem items were scored on eight syndrome scales, two broadband, higher-order subscales (Internalizing and Externalizing Problems), and a Total Problem scale. The Total Problem score was computed by summing the scores for all problem items [50]. The response format of questions on behaviors was 0 = not true, 1 = somewhat or sometimes true, and 2 = very true or often true. The CBCL ratings are based on the child’s functioning over the last six months. An overall good internal consistency of the CBCL/6–18 syndrome scales was reported for the whole sample, i.e., the age group of 6–18 years in a study from Nepal [32]. Acceptable internal consistency was also observed in the adolescent age group targeted in the present study. Cronbach’s alphas for the syndrome scales were 0.72 (Anxious/Depressed), 0.68 (Withdrawn/depressed) 0.77 (Somatic Complaints), 0.67 (Social problems), 0.71 (Thought Problems), 0.77 (Attention Problems), 0.72 (Rule-Breaking Behavior) and 0.86 (Aggressive Behavior).

### Statistical analyses

Statistical analyses were performed using IBM SPSS version 29.0 for Windows [51]. To (1) compare parent perception of difficulties and the impact of problems, and the CBCL Total Problems between three different caste/ethnic groups, to (2) examine the possible moderating effects of caste/ethnicity on the associations between CBCL symptoms and parents’ perception of difficulties and impact of problems, and (3) examine the association between parents’ ratings on the CBCL Total Problems scale and parent perceptions of difficulties and the impact of problems, we employed a mixed model approach to address the hierarchical structure of our data. Our data consisted of adolescents nested within classrooms (Level 2), classrooms nested within schools (Level 3), schools nested within enumerators (Level 4), and enumerators nested within districts (Level 5). This nested structure was modeled using random intercepts at each level to account for the dependencies observed at multiple levels. For continuous outcomes, such as the impact of problems and CBCL problems, we conducted a full 5-level linear mixed model (LMM) analysis, incorporating random intercepts for classrooms, schools, enumerators, and districts. This comprehensive approach allowed us to capture the variability and dependencies across all levels of data hierarchy. For ordinal outcomes, (i.e. perceived difficulties), we utilized a 3-level generalized linear mixed model (GLM), including random intercepts for classrooms and districts. Due to the challenges in estimating variance components at the enumerator and school levels for these ordinal outcomes, these levels were not included in the model. The significance level used in all tests was 0.01.

## Results

Among the study participants (N = 1882), the highest representation was from the Hindu “high caste” group (Brahmin and Chhetri) (48.3%), followed by the indigenous/ethnic minorities group (the Janajati) (40.8%) and the Hindu “low caste” group (Khas Kaami / Dalits) (11.0%). 17.5% of all parents (total sample), perceived their adolescents to have either minor, definite, or severe difficulties in emotions, concentration, behavior, or being able to get on with other people. 21.4% of parents from the “high caste” group, 19.3% from the “low caste” group, and 12.3% from the indigenous/ethnic minorities group perceived either minor, definite, or severe difficulties in their adolescents.

### Parents’ perception of Nepali adolescents’ difficulties–comparison between different caste/ethnic groups

We compared parents’ perceptions of adolescents’ difficulties between the three caste/ethnic groups. We included parents’ perception of adolescents’ difficulties as a dependent variable and added a random effect of district, school, and school class variables while controlling for the living area, parental educational level, father’s employment status, and CBCL Total Problems. We found no significant effect of caste/ethnicity on perceived difficulties (F = 1.14, p = 0.32). Parents from the Hindu “high caste” and “low caste” groups did not differ from parents in the indigenous/ethnic minorities group in their perception of adolescents’ difficulties (Table 1).

**Table 1:** Parents’ perception of Nepali adolescents’ difficulties across different caste/ethnic groups in Nepal (N = 1882)

Caste/ethnic groups	Parent perception of difficulties			
	Fixed coefficient (SE)	t	Odds Ratio	95% Confidence Interval for OR
Reference group: Indigenous/ethnic minorities group (n = 767)	–	–	–	–
Hindu “high caste” group (n = 908)	0.16 (0.19)	0.82	1.17	[0.80, 1.71]
Hindu “low caste” group (n = 207)	− 0.21 (0.28)	− 0.75	0.81	[0.46, 1.41]
Overall effect	F = 1.14, p = 0.32			

*SE* Standard error, *t* t test statistic, *OR* Odds Ratio, *F* F test statistic

### Cross-cultural comparison of the impact of Nepali adolescents’ problems across caste/ethnic groups as reported by their parents

We compared the impact of problems across caste/ethnic groups. We included the Total Impact score as a dependent variable while controlling for the living area, parental educational level, father’s employment status, and CBCL Total Problems. We found no effect of caste/ethnicity on the impact of problems. The “high caste group” and “low caste group” did not differ significantly from the ethnic minority group (Table 2).

**Table 2:** Impact of adolescent difficulties across different caste/ethnic groups in Nepal (N = 1882)

	Estimated marginal means (SE)	Coef. (SE)	t	95% Confidence interval
Reference group: Indigenous/ethnic minorities) (n = 767)	0.43 (0.14)	**–**	**–**	**–**
Hindu “high caste” group (n = 908)	0.44 (0.14)	0.00 (0.06)	0.03	[− 0.12, 0.12]
Hindu “low caste” group (n = 207)	0.49 (0.15)	0.06 (0.09)	0.61	[− 0.13, 0.24]
Overall effect	F = 0.19, p = 0.83			

*Coef*. Regression coefficient, *SE* Standard error, *t* t test statistic, *F* F test statistic

### Nepali parents’ ratings of adolescents’ EBPs on the CBCL Total Problems scale – comparison between different caste/ethnic groups

We compared the mean scores on the CBCL Total Problems for the different caste/ethnic groups while controlling for living area, parental educational level, and father’s employment status. We found that the overall effect of caste/ethnicity was not significant for Total Problems (p = 0.04). However, parents from the Hindu “low caste” group reported higher mean scores than parents from the Indigenous/ethnic minority group (p < 0.01) (Table 3).

**Table 3:** Parent ratings of adolescents’ EBPs on the CBCL Total Problem scale between different caste/ethnic groups in Nepal (N = 1882)

	Total problems			
	Estimated marginal means (SE)	Coef. (SE)	t	95% Confidence Interval
Reference group: Indigenous/ ethnic minorities) (n = 767)	27.66 (2.22)			
Hindu “high caste” group (n = 908)	28.82 (2.19)	1.15 (1.15)	1.00	− 1.11, 3.42
Hindu “low caste” group (n = 207)	32.25 (2.58)	4.59 (1.79)	2.57*	1.08, 8.10
Overall effect	F = 3.31, p = 0.04			

*p < 0.01, *Coef*. Regression coefficient, *SE* Standard Error, *t* t test statistic, *F* F test statistic

### Associations between the CBCL total problems and perceived difficulties and impact of problems

We tested the association between (1) CBCL Total Problems and Perceived Difficulties and (2) CBCL Total Problems and the Total Impact of Problems while controlling for the living area, parental educational level, and father’s employment status. We found a positive association between the Total Impact of problems and the CBCL Total Problems (F = 95.43, p < 0.001). Similarly, perceived difficulties were positively associated with Total Problems (F = 95.72, p < 0.001). However, all associations were only moderately high (Table 4).

**Table 4:** Associations between the CBCL Total Problems and Total Perceived Difficulties and Total Impact of problems

	Impact of problems		Perceived difficulties		
	Coef (SE)	t	Coef (SE)	t	OR
CBCL Total Problems	0.01 (0.00)	9.78**	0.03 (0.00)	9.78**	1.03
Overall effect	F = 95.43**		F = 95.72**		

**p < 0.001, *Coef*. Regression coefficient, *SE* Standard error, *t* t-test statistic, *OR* Odds ratio, *F* F test statistic

### Moderating effects of caste/ethnicity on the associations between the CBCL symptoms and the SDQ impact of problems

We examined whether caste/ethnicity had any moderating effect on the association between (1) CBCL Total Problems and SDQ Perceived Total Difficulties and (2) CBCL Total Problems and SDQ Total Impact of Problems while controlling for living area, parental educational level, and father’s employment status. The moderating effect of caste/ethnicity on the association between CBCL Total Problems and SDQ Perceived Difficulties was not significant (F = 1.57, p = 0.21). Likewise, we found no significant moderating effects of caste/ethnicity on the association between the CBCL Total Problems and the SDQ Total Impact (F = 0.97, p = 0.38).

## Discussion

This study explored parents’ perceptions of their adolescents’ difficulties and the impact of problems between different caste/ethnic groups in Nepal. Further, it examined parent reports on a problem rating scale (the CBCL Total Problems) and the associations between those reports and parents’ perception of difficulties and the impact of problems, taking into consideration the possible moderation effect of caste/ethnicity.

In contrast to findings reported in the literature [9, 15, 52, 53], this study did not find cross-cultural differences in parents’ perceptions of difficulties and the impact of problems between different caste/ethnic groups in Nepal. Our findings may be attributed to various reasons. First, regardless of their caste/ethnicity, Nepali parents might share common attitudes and norms about child behavioral difficulties in the areas investigated in our study which would be consistent with the collectivistic values found in a society such as Nepal [19]. Social mobility, internal migration from rural to urban areas, inter-caste marriages, and subsequent cultural exchanges, as described in recent Nepali papers, might have blurred the lines between different groups of people [54–56], possibly leading to more common norms and more homogeneous parental perceptions. However, such societal mechanisms are complex and their impact on cultural group differences needs to be further explored in future studies to broaden our knowledge of parents’ perception of difficulties and the impact of children’s behavioral problems cross-culturally. Further, the reason behind the null findings may be due to insufficient power rather than the true absence of an effect that needs to be addressed in future studies. Another important reason for non-significant cross-cultural findings may be that the group categories used in this study, i.e., “Hindu high caste” group, “Hindu low caste” group, and “indigenous/ethnic minorities” group were too broad and heterogeneous to capture inter-group differences. For instance, the Janajati group consists of several ethnic groups, such as the Magar, Tharu, Tamang, and Newar, living within different cultural- and social contexts, although all of them live as minorities vis-à-vis the majority Hindus. Furthermore, the caste system itself is complex, with considerable heterogeneity, even within the same caste/ethnic group. Hence, our study required further replication. More detailed and extensive studies with better-defined cultural groups, and with a proper design to capture cultural differences in parents’ perceptions of child difficulties and the impact of problems are warranted.

Finally, Nepali parents’ perception of child difficulties was low across groups, only 17.5% of all parents perceived that their adolescents had either minor, definite, or severe difficulties. Detecting cross-cultural differences in low-prevalence samples is difficult, and non-significant results should be interpreted with caution.

On the other hand, cross-cultural differences in parent ratings on the CBCL Total Problems emerged, with parents from the Hindu “low caste” group reporting more problems. Adolescents in a “low caste” group are likely to be rated higher than other groups on a problem rating scale due to socio-economic adversity / low socio-economic status (SES) and caste-based discrimination prevailing in Nepali society [57] which increases the risk of adverse child mental health outcomes [32, 58–60]. A recent Nepali study suggested that adolescents who have a lot of ecocultural and contextual risk factors and live in unsupervised physical and social settings might have a higher risk of behavioral problems [11]. International studies have shown that socio-economically disadvantaged children and adolescents are at a greater risk of mental health problems [61, 62]. However, more studies are warranted to explore EBPs in “low caste” groups in Nepal.

The moderate association found in our study between CBCL symptoms and parental perceived difficulties, and the impact of problems is consistent with previous international studies [7, 24, 25, 63], suggesting that the parental threshold for describing child behavior as problematic might be different from how they report on measures of symptoms. For instance, Nepali parents might report internalizing behaviors in the CBCL, but at the same time report fewer worries and the impact of such behaviors due to the acceptance of withdrawn or internalizing behaviors in their society. However, future Nepali studies are required to confirm this finding.

Finally, we examined whether caste/ethnicity had a moderating effect on the associations between the CBCL problem scores on the one hand and parents’ perception of difficulties and the impact of problems on the other. When controlling for the living area, parental educational level, and father’s employment status, no moderating effect of caste/ethnicity on any of the above-mentioned associations was found. However, moderation effects are generally difficult to detect and are often small. Future studies with proper design to capture the intricacies inherent within the different cultural groups of Nepal might shed more light on the potential moderation or mediation effects of culture on the associations between symptoms and the impact of problems.

## Limitations

This study has some inherent limitations that should be considered. First, it is important to acknowledge the limited generalizability of our study findings because of the purposive sampling method used to select districts and schools. The use of a probability sampling method in the selection of districts and schools would have enhanced the robustness of the process. Purposive sampling might have resulted in biased results (selection bias). Certain districts or schools may have unique characteristics that influence the results. To overcome these issues, we used a multilevel/mixed method approach in our analyses and controlled for contextual factors such as living area (rural, semi-urban, and urban), parental educational level, and father’s employment status. However, this may only partially adjust to the limitations mentioned above.

Second, perceived difficulties and the impact of problems were exclusively rated from the perspectives of parents which might be considered a limitation of this study. Only 17.5% of parents in the overall sample reported difficulties. Although parents know their children best, they may be biased by their own subjective needs and expectations. Due to the social desirability and stigma of mental disorders in Nepali society [64], parents may have chosen not to communicate their perception of their child’s difficulties and the impact of problems, leading to false negative results. Furthermore, the prevalence of parents’ perception of child difficulties was lowest in the indigenous/minority group (12.3%). One explanation for this could be that some parents belonging to this group might not have properly understood the questions due to low proficiency in the Nepali language and did not communicate this problem to research assistants.

To ascertain parental information, we could have included other research methods, such as additional qualitative interviews or participant observations. However, limited resources have prevented us from doing so. Furthermore, our study did not investigate the effect of recall bias on the CBCL ratings. Ratings on the CBCL are based on parents’ recall of their child’s functioning in the home context over the last six months. Due to the lack of adolescents’ self-reports and teachers’ reports on perceived difficulties and the impact of problems, we could not make a comparison between different informants. Future Nepali studies using multiple informants that report both difficulties and impacts are warranted.

Third, although we measured both problems and the impact of problems in this study, we were unable to identify clinical cases within the present study design. We do not have clinical cut-off scores for Nepal that indicate whether a child’s symptoms are sufficiently severe to be considered a clinical problem (caseness). SDQ norms for Nepal, and norms appropriate to other South-Asian countries have not yet been developed. Preliminary findings suggest that the use of British norms may increase the prevalence rates in these countries [65]. Hopefully, future studies will address this important issue by providing separate SDQ norms for Nepal.

Finally, although we controlled for parental educational level and the father’s employment status in the analyses, this might be inadequate to capture the complexity of the families’ socio-economic status. In future studies, it is recommended to delve deeper into the relationship between cultural- and socio-economic factors and to examine both their influences on parents’ perception of their child’s difficulties and the impact of problems.

## Clinical implications

Cross-cultural knowledge about possible differences and similarities in parents’ perception of adolescents’ difficulties and the impact of problems would be relevant to clinicians when assessing mental health symptoms and treating problems in multicultural societies such as Nepal. Our findings suggest that in their assessments and diagnostic procedures, clinicians should pay attention to the cultural and social context of the child, including parents’ norms and perceptions, rather than focusing solely on children’s symptoms on rating scales. Moderate associations between parents’ rating of symptoms and parents’ reports on the impact of problems suggest that clinicians should be more aware of the possible discrepancies between reported symptoms and functional impairment. Functional impairment is a key factor in determining the clinical importance of mental health problems in children and adolescents. However, assessing impairment has been overlooked by researchers and clinicians. The partial unlinking of symptoms and their impact has implications for clinicians’ prediction of psychiatric disorders, decisions regarding the diagnostic process, and evaluation of treatment [66]. Understanding functional impairments may be particularly important in LMICs, where a better understanding of the social impact of mental health problems on children and adolescents is crucial for clinical services.

## Conclusions

This study examined cross-cultural differences in Nepali parents’ perceptions of their adolescents’ difficulties and the impact of problems. Contrary to the cross-cultural differences found in parent ratings on the CBCL Total Problems scale, we found no differences in parent perception of difficulties and the impact of problems between the caste/ethnic groups used in this study. Furthermore, parent-reported symptoms on the CBCL correlated moderately with parent perceptions and the impact of problems, indicating the need for clinicians to include an assessment of social impairment and parents’ perception of problems in addition to symptoms rated on a symptom rating scale.

## Supplementary Information

Below is the link to the electronic supplementary material.

Supplementary file1 (XLSX 961 KB)

## Data Availability

The datasets used in the present study are provided as supplementary information files.
